# Getting Pabrinex Right: A Multi-cycle Quality Improvement Study Reducing Waste in Thiamine Prescribing at a Scottish District General Hospital

**DOI:** 10.7759/cureus.96322

**Published:** 2025-11-07

**Authors:** Thomas W Noteman, Damien McCoy

**Affiliations:** 1 General Medicine, Inverclyde Royal Hospital, Greenock, GBR

**Keywords:** cost reduction, district general hospital, overprescription, pabrinex, prescriber, prescription, quality improvement, thiamine, waste

## Abstract

Background

Patients with alcohol use disorders (AUD) are at increased risk of Wernicke's encephalopathy (WE) due to thiamine deficiency. At our centre all admitted patients with AUD are assessed for signs, symptoms and risk factors for WE. Such patients are prescribed oral or intravenous (IV) thiamine replacement in accordance with local guidelines; choice of dose and route of administration is determined based on the clinical features present at presentation. Failure to appropriately treat patients with WE may lead to potentially avoidable progression to Korsakoff syndrome. However, unnecessary use of IV formulations, high doses and extended durations of treatment can contribute to unnecessary administrations of IV thiamine, generating financial, environmental and workload costs. In the UK, IV thiamine is usually given in the form of compound vitamin B and C solution (Pabrinex).

Aim

This quality improvement (QI) project aims to quantify and reduce unnecessary prescription of IV Pabrinex in patients with AUD admitted to a district general hospital using a multi-cycle plan-do-study-act (PDSA) methodology.

Materials and methods

This retrospective study involved 106 patients admitted over a four-month period. Two education-based prescriber-targeted interventions were performed. Data collection was performed at baseline and after each intervention. Recommended thiamine dose was calculated by reviewing patients’ admission documentation for presenting signs, symptoms and demographics. These data were then cross-referenced with local guidelines on the management of patients admitted with AUD to determine the recommended thiamine dosing regimen. The total number of IV Pabrinex doses recommended by guidelines was compared to the actual number prescribed for each patient to determine potentially unnecessary “excess” doses. Primary outcome measures recorded were the number of excess doses prescribed and proportion of patients receiving the correct initial dose.

Results

Baseline data showed 60% patients had received excess doses of Pabrinex, with a median of 13 excess doses per patient. This represented an excess cost of £35 per patient for medication alone. Following first and second interventions, median excess doses per patient in subsequent cycles fell to seven and then to 4.5, a significant reduction from baseline (p=0.0087). Proportion of patients receiving correct initial prescription showed a non-significant rise from 30% to 51.3% (p=0.12).

Conclusions

A targeted four-month multi-cycle QI project was carried out to quantify and reduce thiamine overprescription among AUD patients in a Scottish district general hospital. Interventions successfully reduced excess Pabrinex doses given per patient. This study highlights the potential for overprescription in the context of thiamine replacement and findings suggest that low-cost prescriber education strategies can be used to improve practice in this setting. Limitations included the single-centre design and short follow-up period, which limit generalisability. Clinical outcome measures were not recorded here but would be important to consider in future work. Caution should be exercised when aiming to reduce thiamine overprescription to mitigate the risk of underdosing patients who warrant treatment. Following completion of this project, a revised guideline has been implemented for this health board with simplified dosing recommendations, which may yield further improvements in practice.

## Introduction

Patients with alcohol use disorders (AUD) are at increased risk of developing Wernicke's encephalopathy (WE) and its chronic counterpart, Korsakoff syndrome, due to deficiency of vitamin B1 (thiamine) [1]. One recently published study found the incidence of hospitalisations for alcohol-related WE as 5.43 per 100,000 person years, compared with 0.39 per 100,000 person years for non-alcohol-related WE [2]. The most common presenting features of WE secondary to AUD are altered mental status, ophthalmoplegia and gait ataxia, which comprise the classic triad of the disease [3]. Other presenting symptoms reported in the literature include lethargy, seizure, hyporeflexia, limb weakness and aphasia [4]. Local guidelines recommend that all patients with AUD admitted to the hospital be assessed for signs or symptoms of, and risk factors for WE [5], which aligns with current national guidance on the topic [6]. Risk factors listed by local guidelines include weight loss of more than 5% body weight, poor diet, vomiting, alcoholic liver disease or age less than 18 or more than 65 years. Depending on the presence or absence of these factors, the local recommended dosing regimen for thiamine replacement varies; in those with no signs, symptoms or risk factors for the disease the recommended dose is 50mg of oral thiamine four times daily. For those with any risk factors or signs/symptoms of WE, the recommendation is for intravenous replacement with compound vitamin B and C solution (Pabrinex). Among these presentations, total recommended dosing regimen ranges from a 24-hour course comprising three doses and a total of 750mg IV thiamine to a five-day course comprising 21 doses and a total of 5250mg IV thiamine, with the specific features present at presentation determining the choice of regimen [5].

Overprescription is a major source of waste in healthcare [7], and cost control is a key responsibility of physicians, particularly when practicing in publicly funded systems such as the UK National Health Service (NHS) [8]. The unnecessary prescription and administration of intravenous medications such as Pabrinex generates excess costs not only financially, but also in terms of staff workload, environmental impact, nursing burden and risk of medication errors [9-11]. However, there is currently no published data available on the rates of overprescription of thiamine in the inpatient setting. Overpresciption of other drug classes such as opiates and antibiotics has been more extensively studied, with previously published work demonstrating the effectiveness of quality improvement (QI) through prescriber education strategies in these contexts [12,13]. This study aimed to quantify the degree of overprescription of Pabrinex in a Scottish district general hospital when compared to recommended practice. Interventions were then implemented to improve prescribing practice for thiamine replacement in patients with AUD and to reduce the amount of unnecessary prescription of IV Pabrinex.

## Materials and methods

Outcome measures 

Two primary outcome measures were recorded for this study. These were: (i) Number of excess doses of Pabrinex prescribed per patient as compared to guidelines and (ii) Proportion of patients prescribed the appropriate initial dose of thiamine or Pabrinex on admission according to local guidelines. As Pabrinex is supplied in pairs of ampoules, which are mixed to prepare a single dose before administration, one “dose” was defined as one pair of ampoules. One pair of ampules contains 250mg thiamine, 4mg riboflavin, 50mg pyridoxine, 500mg ascorbic acid, 160mg nicotinamide and 1000mg glucose [14].

Data collection and statistical analysis 

Data were collected over three separate periods. These were a five-week baseline data collection period and a five-week and six-week data collection period following the first and second interventions, respectively. During data collection, electronic prescription charts for all patients admitted to medical wards at Inverclyde Royal Hospital were reviewed. For any patients prescribed thiamine replacement therapy, admission documentation was then assessed to confirm whether they had an AUD and, if so, whether there were any documented signs and symptoms of, or risk factors for WE. Inclusion criteria were: (i) admission to a medical ward during the observation period, (ii) prescription of thiamine replacement during the admission and (iii) documentation of a current AUD. Exclusion criteria were (i) prescription of thiamine replacement for a non-alcohol-related indication and (ii) transfer to another hospital site during admission.

Signs, symptoms and risk factors for WE were recorded according to those listed in the relevant treatment guideline [5]. Features recorded as signs or symptoms of WE were any of the following: confusion, ataxia, decreased level of consciousness, ophthalmoplegia, nystagmus, hypothermia or hypotension. Features recorded as risk factors for WE included: weight loss of greater than 5% bodyweight over the past six months, vomiting, reduced oral intake, alcoholic liver disease, age less than 19 or greater than 65 and presence of seizure activity at presentation.

Recommended thiamine or Pabrinex dose and duration were then calculated based on these documented features by reference to the treatment guideline. The total number of doses of Pabrinex or oral thiamine actually prescribed for each patient was recorded from electronic prescription records. The total number of doses given was compared to the recommended dose calculated from admission records to establish the number of “excess” doses prescribed per patient.

Similarly, the recommended initial dose of thiamine on admission was determined based on the documented features of or risk factors for WE on admission, and this was compared to the actual initial dose prescribed on electronic medical records to determine whether each patient had received the correct initial dose.

Significance testing between data collection periods was carried out for each of the outcome measures. For data on the number of excess doses given per patient, testing for normality of distribution was first performed using a Shapiro-Wilk test, which showed a significant departure from normal distribution (p<0.001), meaning that the assumptions required for parametric testing methods were not met. Differences between groups were therefore assessed for significance using the non-parametric Kruskal-Wallis test and post-hoc Dunn's test using Bonferroni correction for multiple tests. For data on the proportion of patients in whom the appropriate dose of thiamine or Pabrinex had been prescribed, and on the proportion of patients receiving fewer doses than recommended, chi-squared tests were used with Bonferroni correction for multiple tests. Comparison between patient characteristics in the three data collection periods was performed using chi-squared tests. Statistical testing was carried out using GraphPad Prism software (La Jolla, CA, USA).

Interventions 

**Figure 1:**
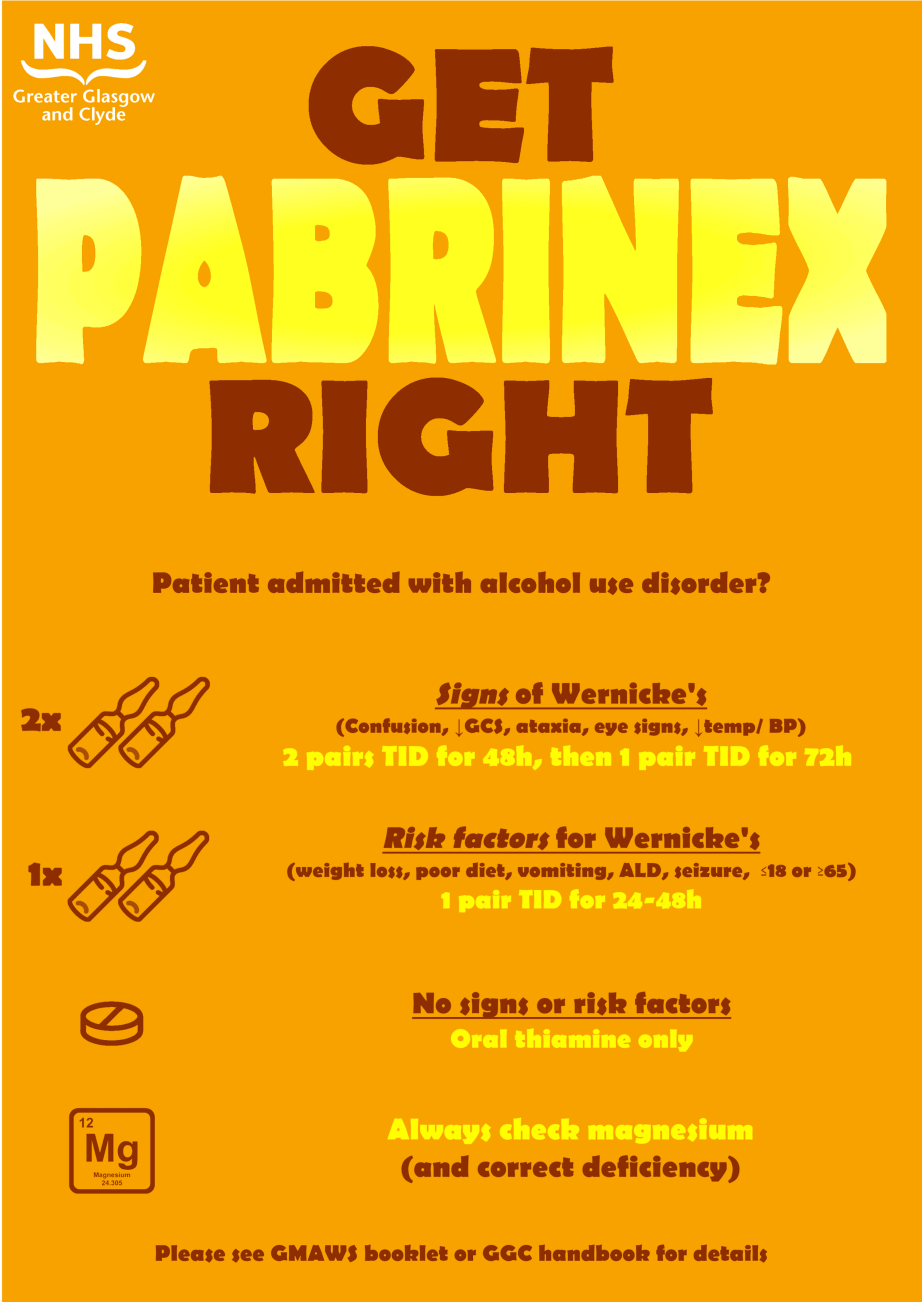
Infographic-style poster developed during the first intervention cycle of this project. Image credits: T Noteman. Information presented is based on regional guideline [5].

Following the five-week baseline data collection period in March-April 2024 and review of this data, the first intervention was implemented in May 2024. An infographic-style chart summarising the guidelines for recommended dosage and duration of thiamine replacement in patients admitted with AUD was developed (see Figure 1). This was circulated via email to prescribers working in the medical admissions team and displayed in the doctors’ office on the acute medical unit. A further five-week data collection and analysis period immediately followed this intervention.

The week following this second data collection period, the second intervention was implemented, consisting of teaching sessions provided to resident and consultant doctors in the hospital to highlight local prescribing guidelines and the extent to which current practice deviated from this. Financial, environmental and workload costs of overprescribing were highlighted. Although consultant doctors are not routinely responsible for prescribing, it was hoped that their involvement would support more sustained improvements through longer-term oversight of practice in the unit. Data were then collected for a final six-week period immediately following the second intervention.

Patient characteristics 

Across all data collection periods, 106 patients met inclusion criteria, with 40, 27 and 39 patients included in each round of data collection respectively. The age of included patients ranged from 24 to 81 years, with a mean of 50.8 years. Patients included were 69.8% male (74/106) and 30.1% female (32/106).

Patients displaying signs or symptoms of potential WE represented 11 of 40 patients (27.5%) in the baseline period, seven of 27 (25.9%) in the first post-intervention period, and 10 of 39 (25.6%) in the second post-intervention period. Patients with identified risk factors for WE but who displayed no signs or symptoms represented 24 of 40 patients (60%) in the baseline period, 14 of 27 (51.9%) in the first post-intervention period and 25 of 39 (64.1%) in the second post-intervention period. Patients with no signs, symptoms or risk factors for WE represented five of 40 patients (12.5%) in the baseline period, six of 27 (22.2%) in the first post-intervention period and four of 39 (10.3%) in the second post-intervention period. There was no statistical difference in the distribution of patients in these three categories across the three data collection periods (X2 = 2.1735, p = 0.70).

## Results

Outcome measures 

Excess Pabrinex Doses 

**Figure 2:**
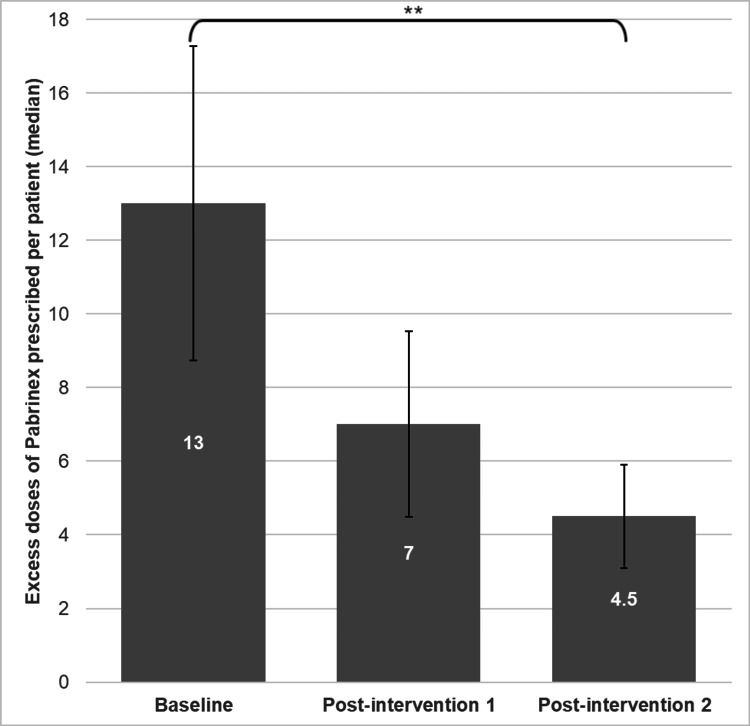
Median excess doses (pairs of vials) of Pabrinex prescribed per patient in the baseline, post-intervention 1 and post-intervention 2 data collection periods Our baseline data showed a median of 13 excess doses prescribed per patient. Following one intervention this fell to seven and after the second intervention fell further to 3.5. ** p-value <0.01. Error bars show standard error of data for each collection period.

**Figure 3:**
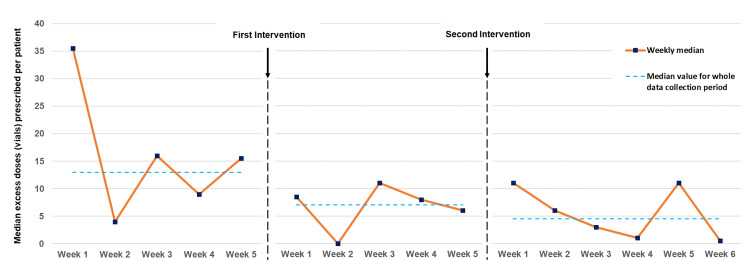
Weekly run chart showing median excess Pabrinex doses (pairs of vials) prescribed per patient in the baseline, post-intervention 1 and post-intervention 2 data collection periods Following interventions, there was evidence of a reduction in excess Pabrinex prescribing. At baseline, a weekly median excess Pabrinex doses prescribed per patient of >15 was observed in three weeks out of five. In the post-intervention periods, weekly median did not surpass 11 at any point.

Excess doses of Pabrinex prescribed per patient fell from a median of 13 to 7 and then to 4.5 across the three of eight data collection periods (Figures 2, 3). This represented a 65.4% reduction over the course of the study and was highly statistically significant (p=0.0087). There were non-significant reductions in excess doses prescribed between the first and second (p=0.409) and second and third (p=0.14) data collection periods.

Correct Initial Prescription

**Figure 4:**
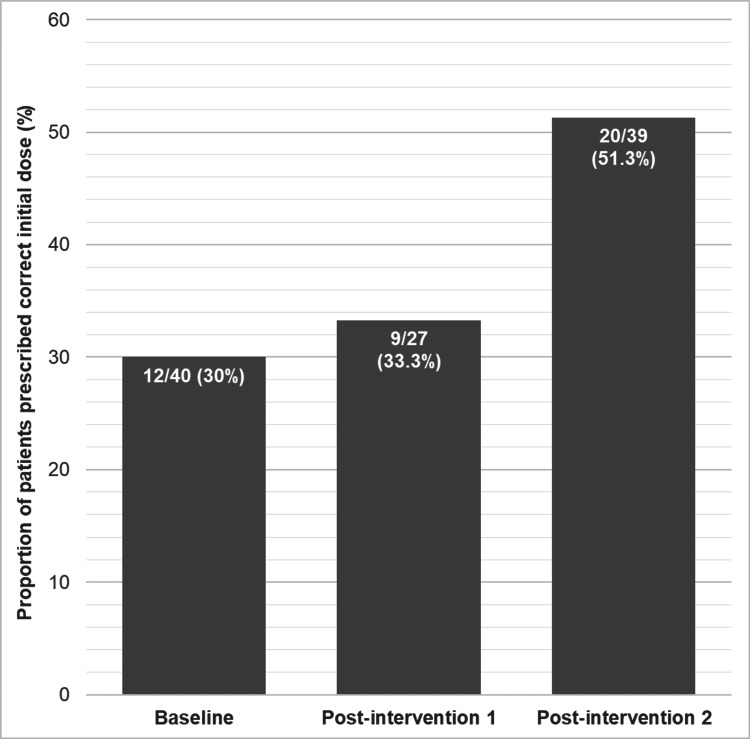
Proportion of patient prescribed he correct initial dose of thiamine replacement according to local guidelines in the baseline, post-intervention 1 and post-intervention 2 data collection periods Proportion of patient prescribed correct initial dose of thiamine rose from 12/40 (30%) at baseline, to 9/27 (33%) after first intervention and 20/39 (51.3%) after intervention 2. There was no statistically significant difference between the three groups (X^2^=4.2074, p=0.12)

The proportion of patients prescribed the appropriate initial dose of thiamine or Pabrinex increased from 12 of 40 (30%) to nine of 27 (33.3%) and to 20 of 39 (51.3%) over the three data collection periods (Figure 4). These differences between groups however were not statistically significant (X2=4.2074, p=0.12). Odds ratio of receiving a correct prescription following the second intervention as compared to baseline was 2.45 (95% confidence intervals 0.90-6.67).

Underdosing of patients 

As a balancing measure, the number of patients who received fewer doses of Pabrinex than would be recommended by guidelines was also recorded. This did not change significantly throughout the study period. This proportion was 12 of 40 (30%), 10 of 27 (37.0%) and 10 of 39 (25.6%) across the baseline, first post-intervention and second post-intervention data collection periods respectively. There was no statistically significant difference among these three groups (X2=0.9842, p=0.61).

## Discussion

Principle findings 

This multi-cycle quality improvement project demonstrated a significant improvement in the first of its primary outcome measures, the number of excess doses of Pabrinex prescribed per patient when compared to local guidelines. In the second main outcome measure, proportion of patients prescribed the correct initial dose of thiamine or Pabrinex on admission, a statistically non-significant improvement was observed. This study suggests that low-cost educational interventions can be used to achieve improved accuracy in prescribing practice and considerable reductions in overprescribing over a short time period in the district general hospital setting. In clinical terms, the reduction in excess doses observed reduces the opportunity for medication errors during administration, minimises patient exposure to potentially unnecessary IV treatments and optimises use of resources both in terms of materials and staff workload. Additionally, to consider the health economics of this change, at the currently listed NHS indicative price of Pabrinex of £16.23 for 12 ampoules (six doses) [15], and usage levels observed during this project, the reduction in excess prescribing demonstrated here if sustained in the longer term could save this hospital approximately one thousand pounds per month in medication costs alone.

While the number of excess doses per patient fell significantly, the proportion of patients prescribed the correct initial dose showed a more modest and statistically non-significant rise from 30% to 51.3%. The discrepancy between the degree of change in the two outcome measures suggests that the reduction in number of excess doses of Pabrinex prescribed could in part be due to higher rates of review and correction of initially incorrect doses following initial admission.

Context within existing literature 

Overprescribing is a recognised problem within the UK NHS; overprescribing in NHS England was the subject of a recent review by the UK government’s chief pharmaceutical officer, which estimated that at least 10% of primary care prescriptions were unnecessary [16]. Published evidence on overprescribing generally in inpatient settings is limited, with published literature largely focusing on specific drug classes such as opiates or antibiotics. Previous quality improvement work in these areas has however demonstrated the potential for prescriber education strategies to reduce overprescription rates [12,13]. Our findings align with previous work on prescribing practices in relation to thiamine replacement for AUD, which has shown that non-adherence to recommended prescription regimens is a significant issue [17,18]. Previous work on thiamine prescription has focused on the clinically important issue of under-prescription and how implementation of electronic decision support measures can improve practice in this regard [19]. We have identified evidence of overprescribing as a challenge which also warrants attention and has not been addressed previously. To our knowledge this is the first published work quantifying the degree of thiamine prescription, making it a valuable addition to the literature. As our findings are in one district general hospital, it remains uncertain to what extent the findings presented here are representative of practice more broadly. The appropriate dosing regimen of thiamine replacement for the prevention and treatment of WE is a topic on which there is not a strong evidence base; published dosing strategies vary in drug dose, duration and route of delivery, without conclusive evidence for an optimal schedule [20]. While there is no nationally or internationally recognised optimal dosing regimen published for thiamine replacement, the dosing recommendations of the relevant guideline to this study were broadly in line with those published elsewhere, suggesting thiamine replacement doses of 250mg or 500mg for one to five days depending on the patient’s signs, symptoms and risk factors [1,5]. Following completion of this project, the guideline issued by this health board has been updated, simplifying the suggested dosing regimen for patients at risk of WE [21]. Whether this change influences prescribing practice in thiamine replacement will be an interesting topic for future work.

Implications for practice 

This project highlighted a need for increased accuracy in prescribing practice of thiamine replacement at a district general hospital. The degree to which the education-based interventions described here were successful in achieving this aim supports the hypothesis that a lack of knowledge of current guidelines among prescribers was a major factor in erroneous prescribing practice. While local guidelines are available via the intranet and in paper form in all admissions areas, lack of awareness of these guidelines' availability could lead to their not being adhered to. Raising awareness of these guidelines was therefore a key aim of our interventions. Other reasons for lack of adherence are likely to include the relative complexity of the guideline in use at the time of the study, which recommended four possible dosing regimens for thiamine replacement based on patient factors. Differing levels of experience among prescribers may contribute; although we did not differentiate grade of prescriber in this project, differences in practice between prescribers with different levels of experience would be an interesting topic to explore. The lack of a consensus around optimal thiamine dosing schedule, leading to regional and national variability in practice is also likely to contribute to variability in practice, as prescribers moving between regions in their training may not be up to date with differing local guidelines. In this context prescribers may default to prescribing high-dose thiamine replacement for all patients with AUD, leading to potentially unnecessary prescriptions.

It is interesting to consider why the proportion of patients being prescribed the correct initial dose of thiamine remained relatively low at 51.3% even after two intervention cycles in this study. Given that admissions are typically unplanned and can occur at any time, a significant proportion of initial prescriptions are completed out of hours and at times of high workload - both factors which are known to reduce prescribing accuracy [22,23]. Diagnostic uncertainty is also likely to be a factor, given that some features of acute alcohol intoxication can resemble WE on first presentation [24]. Prescribers completing initial admission prescriptions are therefore potentially acting under conditions of lower diagnostic certainty and of higher cognitive load when compared to post-admission reviews. Under such conditions it may be hypothesised that prescribers may therefore tend to err on the side of caution in prescribing high-dose thiamine replacement, with a proportion of these prescriptions subsequently being corrected on post-admission review. Data collected here were not sufficient to provide evidence for or against this hypothesis. 

While it is true that thiamine replacement therapy has very limited adverse effects and its prescription may therefore be perceived as “risk-free" [25,26], financial, environmental and workload costs should also bear consideration in prescribing decisions. Part of the aim of the second intervention cycle in this study was to raise prescriber awareness of these factors. Further proposed methods to address issues in prescribing practice identified here include simplification and standardisation of guidelines, integration of guidelines with electronic prescribing systems in the form of decision support tools or automated alerts to prescribers. These changes were beyond the scope of those that could be implemented in the timeframe of this study but could be valuable systems-level interventions. However, this project has successfully demonstrated that low-cost, simple interventions aimed at improving the knowledge of prescribers can yield significant improvements in a short period of time. Sustaining the changes shown through annual cycles of staff turnover will likely require a continuation of this QI work. 

Strengths and limitations 

A key strength of this study is its real-world clinical relevance. It focused on a clearly defined patient population and directly evaluated adherence to published local guidelines, allowing for actionable insights with immediate applicability. The use of a multi-cycle plan-do-study-act (PDSA) design with sequential interventions enabled assessment of changes over time and supported iterative improvement. Interventions were implemented without incurring costs of additional staff training or changes to current systems of prescribing, making these cost-effective and easily replicable in other settings. Additionally, the inclusion of a balancing measure helped ensure that reductions in overprescribing did not result in patient undertreatment. The reduction in median number of excess Pabrinex doses prescribed per patient achieved was substantial, falling by approximately two-thirds from 13 to 4.5 after two interventions, a reduction which was highly statistically significant.

The study does, however, have several limitations. It was conducted at a single district general hospital, which may limit generalisability of findings to other settings with different patient populations or prescribing cultures. The relatively small size of the centre could mean that educational measures were able to quickly change prescribing culture in a way which would not be so easily replicable at larger sites. Data collection relied on documentation in medical records, which if incomplete or inconsistent, could lead to misclassification of patients’ risk status, and the use of retrospective data introduces risk of documentation bias. Furthermore, clinical outcomes such as duration of symptoms and length of stay were beyond the scope of this project, meaning that the impact of prescribing changes on patient outcomes was not evaluated. Lastly, the relatively short post-intervention follow-up period leaves open the question of how sustainable these changes will prove to be. Despite these limitations, this study can offer a practical model for reducing unnecessary prescribing and highlights the value of pragmatic, low-cost interventions in improving prescribing practice.

Future research 

Future research should investigate whether the recent simplification of prescribing guidelines for thiamine replacement has had an impact on practice, as this was not captured during this study. Furthermore, it would be valuable to repeat similar work to compare practice across several hospital sites to investigate differences in practice regionally and establish the broader applicability of this work. Where interventions are implemented, longer-term follow-up to assess the sustainability of changes in practice would be valuable, as would recording and analysis of actual cost savings in practice where such interventions are implemented.

The increasing availability of artificial intelligence (AI) tools could also be incorporated into future work in this area to aid in data collection, analysis, and monitoring. For example, AI-driven clinical decision support systems could track prescribing behaviours across multiple hospital sites, identify patterns of non-compliance, and predict where guideline simplification might have the greatest impact. This would allow for real-time cost-saving analyses and predictive modelling of resource utilization. By combining traditional follow-up methods with AI-enabled analytics, future studies could achieve a higher level of precision, scalability, and applicability, ultimately ensuring that practice changes are both sustainable and evidence-based [27]. This would however require appropriate AI tools to be approved and validated for clinical research within the NHS and with robust safeguards in place to ensure data security where such AI tools would be using patient data.

## Conclusions

This multi-cycle quality improvement project significantly reduced overprescription of thiamine replacement therapy for patients with AUD in a Scottish district general hospital. For the first time in the literature we have quantified overprescription of thiamine in the setting of inpatients admitted with AUD. Interventions were clinician-led and focused on educating prescribers on current guidelines as well as the potential financial, environmental and workload consequences of overprescribing. Work to simplify and standardise guidelines for thiamine replacement therapy more widely could be beneficial to reduce uncertainty among prescribers about recommended practice. Limitations of this study included its single-centre design and relatively short follow-up period, which raised questions regarding the sustainability of the changes observed and the generalisability of these findings to other settings. Future research should focus on evaluating the long-term sustainability of these interventions and exploring whether similar strategies can be implemented across other hospital settings. Importantly, undertreatment for patients at risk of WE remains a risk which should be considered in any future work aiming to reduce overprescription in thiamine replacement. Overall, this study’s findings suggest that low-cost prescriber education strategies can be successfully implemented in a district general hospital to meaningfully improve practice and improve adherence to regional guidelines.

## References

[1] Latt N, Dore G (2014). Thiamine in the treatment of Wernicke encephalopathy in patients with alcohol use disorders. Intern Med J.

[2] Rasiah R, Gregoriano C, Mueller B, Kutz A, Schuetz P (2024). Hospital outcomes in medical patients with alcohol-related and non-alcohol-related Wernicke encephalopathy. Mayo Clin Proc.

[3] Sinha S, Kataria A, Kolla BP, Thusius N, Loukianova LL (2019). Wernicke encephalopathy-clinical pearls. Mayo Clin Proc.

[4] Cantu-Weinstein A, Branning R, Alamir M, Weleff J, Do M, Nero N, Anand A (2024). Diagnosis and treatment of Wernicke's encephalopathy: a systematic literature review. Gen Hosp Psychiatry.

[5] (2025). Glasgow assessment and management of alcohol (GMAWS) adult inpatients GGC. https://rightdecisions.scot.nhs.uk/media/2688/id-137-gmaws-adult-inpatients.pdf.

[6] (2025). Alcohol-use disorders: diagnosis and clinical management of alcohol-related physical complications. https://www.nice.org.uk/guidance/cg100/chapter/Recommendations.

[7] Olivares-Tirado P, Zanga R (2023). Waste in health care spending: a scoping review. Int J Healthc Manag.

[8] (2025). Domain 1: knowledge, skills and development. https://www.gmc-uk.org/professional-standards/the-professional-standards/good-medical-practice/domain-1-knowledge--skills-and-development#managing-resources-effectively-and-sustainably-474B942EE92A4190B5B7912096698BF3.

[9] Cyriac JM, James E (2014). Switch over from intravenous to oral therapy: a concise overview. J Pharmacol Pharmacother.

[10] Lipkin NC (2012). The environmental impact of health care: implications for infusion nursing. J Infus Nurs.

[11] Keers RN, Williams SD, Cooke J, Ashcroft DM (2015). Understanding the causes of intravenous medication administration errors in hospitals: a qualitative critical incident study. BMJ Open.

[12] Bivacca K, Nadeau C (2023). Quality improvement project for antibiotic stewardship in urgent care. J Nurse Pract.

[13] Kaley A, Brenner JM, Prince AM, Wojcik SM (2023). Effect of a targeted quality improvement education on opioid prescribing. BMJ Open Qual.

[14] (2025). Pabrinex/vitamin B&C intravenous high potency, concentrate for solution for infusion. https://www.medicines.org.uk/emc/product/1427/smpc.

[15] (2025). Vitamin B substances with ascorbic acid, Medicinal forms. https://bnf.nice.org.uk/drugs/vitamin-b-substances-with-ascorbic-acid/medicinal-forms/.

[16] (2025). Good for you, good for us, good for everybody—a plan to reduce overprescribing to make patient care better and safer, support the NHS, and reduce carbon emissions. https://assets.publishing.service.gov.uk/media/614a10fed3bf7f05ab786551/good-for-you-good-for-us-good-for-everybody.pdf.

[17] Thomson AD, Marshall EJ, Bell D (2013). Time to act on the inadequate management of Wernicke's encephalopathy in the UK. Alcohol Alcohol.

[18] Day E, Callaghan R, Kuruvilla T (2010). Pharmacy-based intervention in Wernicke's encephalopathy. Psychiatrist.

[19] Baron SW, Wai JM, Aloezos C, Cregin R, Ceresnak J, Dekhtyar J, Southern WN (2024). Improving thiamine prescribing in alcohol use disorder using electronic decision support in a large urban academic medical center: a pre-post intervention study. J Subst Use Addict Treat.

[20] Smith H, McCoy M, Varughese K, Reinert JP (2021). Thiamine dosing for the treatment of alcohol-induced Wernicke’s encephalopathy: a review of the literature. J Pharm Technol.

[21] (2025). Glasgow assessment and management of alcohol (GMAWS) adult inpatients (137). https://rightdecisions.scot.nhs.uk/media/wjppc5nk/137-gmaws-v7.pdf.

[22] Hendey GW, Barth BE, Soliz T (2005). Overnight and postcall errors in medication orders. Acad Emerg Med.

[23] Leviatan I, Oberman B, Zimlichman E, Stein GY (2021). Associations of physicians' prescribing experience, work hours, and workload with prescription errors. J Am Med Inform Assoc.

[24] Eva L, Brehar FM, Florian IA (2023). Neuropsychiatric and neuropsychological aspects of alcohol-related cognitive disorders: an in-depth review of Wernicke’s encephalopathy and Korsakoff’s syndrome. J Clin Med.

[25] Wrenn KD, Murphy F, Slovis CM (1989). A toxicity study of parenteral thiamine hydrochloride. Ann Emerg Med.

[26] Nishimoto A, Usery J, Winton JC, Twilla J (2017). High-dose parenteral thiamine in treatment of Wernicke’s encephalopathy: case series and review of the literature. In Vivo.

[27] Ogut E (2025). Artificial intelligence in clinical medicine: challenges across diagnostic imaging, clinical decision support, surgery, pathology, and drug discovery. Clin Pract.

